# Mechanism of reduced intestinal phosphate absorption by tenapanor: a hypothesis

**DOI:** 10.1093/ckj/sfaf375

**Published:** 2025-12-01

**Authors:** Takeshi Nakanishi, Tilman B Drueke, Takahiro Kuragano

**Affiliations:** Division of Kidney, Dialysis and Cardiology, Department of Internal Medicine, Hyogo Medical University, Nishinomiya, Hyogo, Japan; Department of Nephrology, Gojinkai Sumiyoshigawa Hospital, Kobe, Hyogo, Japan; Inserm Unit 1018, Team 5, CESP, Hôpital Paul Brousse, Paris-Saclay University (UPS) and Versailles Saint-Quentin-en-Yvelines University (UVSQ), Villejuif, France; Division of Kidney, Dialysis and Cardiology, Department of Internal Medicine, Hyogo Medical University, Nishinomiya, Hyogo, Japan

**Keywords:** NHE3 inhibitor, phosphate, paracellular pathway, tight junction, negative pore charge

## Abstract

Tenapanor, a selective inhibitor of the sodium/hydrogen exchanger isoform 3 (NHE3), was initially developed for the treatment of irritable bowel syndrome with constipation. Subsequent preclinical and clinical studies revealed its ability to reduce gastrointestinal phosphate absorption, leading to effective serum phosphate control with minimal pill burden in patients with kidney failure undergoing dialysis therapy. However, the precise mechanisms underlying NHE3 inhibition, its impact on phosphate handling and the primary site of action within the gastrointestinal tract remain incompletely understood. This review explores the hypothesis that tenapanor-induced NHE3 inhibition elevates the luminal pH via enhanced bicarbonate secretion in the colon, thereby altering phosphate speciation. Phosphate exists in the body as monovalent (H₂PO₄⁻) and divalent (HPO₄²⁻) anions, with the latter predominating under alkaline conditions. Although divalent anions are theoretically more prone to be absorbed from the gut lumen via the paracellular transport route because of the lumen-negative transepithelial potential, on the contrary recent studies have provided evidence that monovalent species are transported more efficiently and that paracellular phosphate permeability is suppressed at high luminal pH. We now propose that the net negative electrostatic environment within the paracellular pore pathway of tight junctions may selectively hinder divalent phosphate transport. This hypothesis aligns with prior findings that tenapanor does not alter the expression of tight junction proteins, suggesting a physicochemical rather than a structural basis for reduced permeability. Further investigations are warranted to determine whether the electrostatic properties of the paracellular pathway contribute to the phosphate-lowering effect of tenapanor.

## INTRODUCTION

Phosphorus is an indispensable nutrient for all living organisms and plays crucial roles in various biological processes, including cell energy metabolism, synthesis of nucleic acids, membrane integrity and skeletal mineralization. In patients with chronic kidney disease (CKD), renal excretion of phosphate is reduced as glomerular filtration decreases, most notably and to an extreme extent in patients on haemodialysis. Excessive retention of phosphate in the body is toxic and causes various cellular and tissue injuries associated with changes in fibroblast growth factor-23, αKlotho, calcitriol and parathyroid hormone metabolism, vascular calcification, premature aging and early mortality [1–3].

To improve patient outcomes by preventing phosphate retention, clinical guidelines advise maintaining normal serum phosphate levels in individuals with CKD, although the recommended upper limit for serum phosphate varies slightly across guidelines worldwide [4–6]. The primary source of phosphorus intake is diet, including food, food additives and drinks. In addition to the removal of phosphate by dialysis therapy, a low-phosphorus diet and phosphate binder use are usually unable to consistently achieve and maintain serum phosphate concentrations near normal despite being recommended for many patients on dialysis therapy. Phosphate binders, i.e. drugs creating nonabsorbable phosphate-containing compounds in the gastrointestinal tract that are excreted in the stool, have long been the only available pharmacological therapy for hyperphosphatemia [7]. Outside the controlled setting of clinical trials, real-world chart audits have revealed that most patients on dialysis receiving phosphate binders struggle to maintain serum phosphate levels consistently at or below 5.5 mg/dL over a 6-month period [7]. These findings indicate that current approaches to phosphate management are often inadequate for counterbalancing the phosphorus load from daily dietary intake. The major factors causing the above are that the daily pill burden in patients on dialysis is one of the highest reported to date in any chronic disease state [8]. A high pill burden is associated with poor health-related quality of life and certainly causes low treatment adherence [8].

Tenapanor hydrochloride (hereafter referred to as ‘tenapanor’) is a first-in-class, minimally absorbed, small-molecule inhibitor of gastrointestinal sodium/hydrogen (Na^+^/H^+^) exchanger isoform 3 (NHE3, SCL9A3) for the treatment of irritable bowel syndrome with predominant constipation (IBS-C) in adults [9]. It has been shown to reduce paracellular phosphate transport and facilitate the management of hyperphosphatemia [3, 10]. The inhibition of phosphate absorption by a novel therapeutic class targeting this pathway provides a new mechanistic understanding of phosphate absorption from the gut lumen [3].

A mechanism has been proposed through which the inhibition of intestinal sodium absorption by NHE3 is accompanied by reduced phosphate absorption but this mechanism has not yet been definitively established. Clearly, tenapanor inhibits NHE3 throughout the gastrointestinal tract, but it is unclear in which intestinal segment the decrease in phosphate absorption mainly occurs. Moreover, electrolytes, including phosphate, are not only absorbed from, but also secreted into the digestive tract, particularly in the small intestine. The final amount excreted in the faeces is controlled by the distal portion of the colon.

In this review, we first outline present knowledge of intestinal phosphate absorption and its inhibition by tenapanor, and then propose a novel mechanism of action of this drug.

## DEVELOPMENT OF AN INHIBITOR OF NHE3

**Figure 1:**
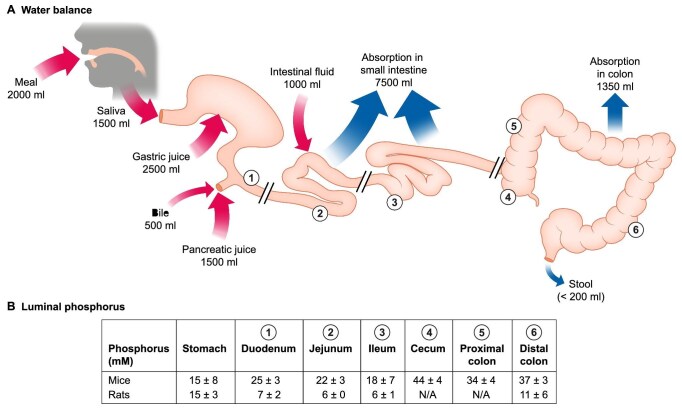
Overall water balance and luminal phosphate concentration in the gastrointestinal tract. (**A**) Water balance: the gastrointestinal tract is exposed each day to approximately 1500–2000 mL of ingested fluid plus 7000 mL of secretions through the mucosa into the gastrointestinal lumen and from associated glands. Ninety-eight percent of the fluid is reabsorbed: 83% in the small intestine and 15% in the colon. The daily fluid loss with the stools is <200 mL. The movement of sodium (Na) in and out of the digestive tract is generally estimated to be similar to that of water. (**B**) Luminal phosphate concentrations in rodents consuming a standard diet (mice and rats). The phosphate concentrations in the intestinal lumen of mice were taken as integer values from the graph presented in reference [30], and those of rats from reference [32].

The idea behind tenapanor development was that promoting gastrointestinal motility by increasing intestinal salt and fluid content could be a viable approach for treating patients with constipation-related disorders [11]. To explain the background of drug development it is worth noting that the gastrointestinal system exhibits considerable complexity, and the breadth of content it encompasses is substantial [12]. Every day, the gastrointestinal tract processes 8–10 L of water and approximately 800 mEq of Na. The majority of the water and sodium (∼7.5 L of water and ∼650 mEq of Na^+^) is secreted into the gastrointestinal lumen as part of the digestion (i.e. only a small portion of the water, ∼1.5 L, and the Na^+^, ∼150 mEq, is ingested) [12, 13]. The largest amounts of NaCl and water are absorbed in the small intestine, whereas in the large intestine smaller additional amounts are absorbed as appropriate to obtain a soft yet well-formed stool (Fig. 1).

Among the different transporters involved in Na^+^ and Cl⁻ absorption, *NHE3* is abundantly expressed all along the gastrointestinal tract and contributes significantly to the transport process. As regards the distribution of *NHE3* expression the intestinal tract of rats, mice, rabbits and humans, it varies by species and gut segment [13–17]. A screening of the *NHE3* gene expression profile in several rat organs revealed higher expression in the stomach, whole small intestine and colon than in the kidney [18]. Based on this observation, NHE3 inhibitors were considered to be potentially effective for treating IBS-C, and this led to the development of tenapanor. Despite the expanding clinical use of tenapanor, the exact nature of this drug and its role in the intestinal absorption of several solutes, including phosphate, remain poorly defined.

## DISCOVERY OF THE ROLE OF TENAPANOR IN PHOSPHATE AND POTASSIUM TRANSPORT

Tenapanor has been reported to reduce intestinal phosphate absorption in both rats [19] and healthy volunteers [20]. Subsequently, clinical trials have shown that tenapanor reduces serum phosphate levels in patients on dialysis with hyperphosphatemia by reducing intestinal phosphate absorption [21]. Generally, no serious adverse effects were observed in the clinical trials conducted for drug approval; only diarrhoea was noted which ceased upon drug discontinuation [21–23]. Most importantly, the administration of tenapanor was associated with a reduction in phosphate binder pill burden, which is expected to improve drug treatment adherence [24, 25]. Consequently, tenapanor was approved as a treatment for hyperphosphatemia in patients with end-stage kidney disease in Japan and the USA. At present, tenapanor is considered to have a relatively good safety and tolerability profile.

As regards intestinal potassium transport, the effect of tenapanor remains unclear. Its administration to rodents led to a decrease in cecal luminal potassium concentration compared with control animals, but did not modify urinary potassium excretion [10]. Studies in homozygous mutant Slc9a3^–/–^ mice and intestinal epithelial cell-specific *NHE3*-KO mice revealed an increase in plasma potassium levels compared with controls [26, 27]. Furthermore, an induction of mRNA encoding colonic H^+^, K^+^-ATPase (cHKA) was observed in Slc9a3^–/–^ mice. It was hypothesized that cHKA might play a critical role in K^+^ conservation, as evidenced by the observation that it mediates the recovery of K^+^ that is secreted during electrogenic Na^+^ reabsorption [28]. As cHKA also secretes H^+^, the concerted activities of ENaC, the K^+^ channel and cHKA may mediate Na^+^/H^+^ exchange [26]. However, in a multicentre, open-label, single-arm study tenapanor did not affect serum potassium in patients on haemodialysis therapy [24], and the absence of changes in serum potassium was confirmed in a recent meta-analysis [29]. Differences in the degree of NHE3 inhibition by tenapanor versus genetic ablation and the role of other regulatory mechanisms of potassium balance might explain apparently discrepant findings between animal and human studies.

## PHOSPHATE TRANSPORT IN THE INTESTINE

There is abundant experimental evidence in humans and rodents in favour of at least two pathways for the absorption of phosphate: active transcellular phosphate transport via a Na^+^-dependent phosphate transporters and the paracellular pathway involving interepithelial tight junctions [30–33].

**Figure 2:**
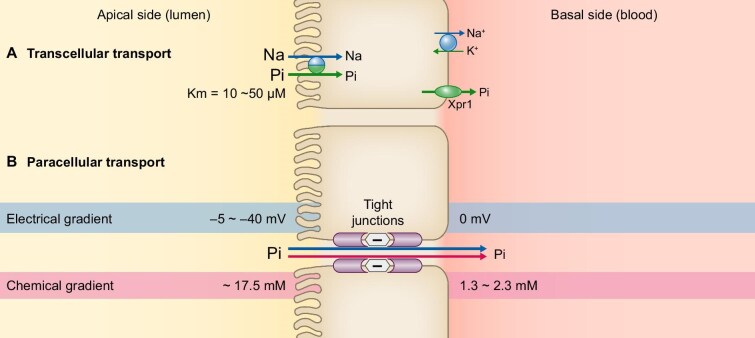
Phosphate transport in the intestine. (A) Transcellular transport: transcellular phosphate (Pi) absorption depends on a secondary active transport mechanism that involves the concentration gradient of Na⁺ from the gut lumen to the enterocyte cytoplasm created by the activity of Na⁺/K⁺ ATPase located at the serosal (basolateral) side. The apical transporter that is mainly involved in this process is NaPi-IIb. However, since its Km has been reported to be as low as 10–50 μM, NaPi-IIb is assumed to function effectively only at very low luminal phosphate concentrations. Depicted are the Na/Pi contransporter (NaPi-II) on the apical cell membrane and the Na⁺/K⁺ ATPase and Xpr1 (potential phosphate exit pathway) on the basolateral cell membrane. (B) Paracellular transport: paracellular phosphate absorption is its movement across the tight junctions between intestinal epithelial cells and is thought to occur via the electrochemical potential and the chemical gradient from the luminal to the serosal side of the gastrointestinal wall. It is hypothesized that both the negative electrical gradient of the lumen (blue arrow) and the positive chemical gradient (red arrow) through the intercellular spaces, directed from the luminal toward the serosal side, promote phosphate absorption.

Regarding the active transcellular phosphate transport, two families of sodium-dependent phosphate transporters, type II [NaPi-IIb (Slc34a2)] and type III (SLC20), are responsible for phosphate import from the gut lumen (Fig. 2A) [34–36]. The type III transporters, which include Pit1 and Pit2, are expressed broadly across a variety of cell types, but are believed to play only a minor role in total phosphate uptake [37]. They are generally considered to be involved in supplying cells with inorganic phosphorus to meet the needs of individual cell functions [34]. NaPi-IIb is the major transporter responsible for active and saturable transcellular phosphate transport in the intestine. However, it has been demonstrated that in CKD rats the contribution of NaPi-IIb to total intestinal phosphate absorption is even lower than normal, whereas low-affinity transporters such as PiT-1 gain in importance [38].

Phosphate absorption involving the NaPi-IIb cotransporter mainly occurs when luminal phosphate concentration is low. Since generally its concentration is much higher than the apparent Km of NaPi-IIb for phosphate (10–50 μM), the actual transcellular transport rate is far lower than that across the paracellular pathway (Fig. 2A) [39, 40].

An additional mechanism is required for phosphate export from the basolateral side to the blood compartment. XPR1 (Xenotropic and Polytropic Retrovirus Receptor 1) is thought to primarily serve as the phosphate export transporter (Fig. 2A). However, exact details regarding its function remain to be further clarified [34].

Regarding the effect of tenapanor on transcellular phosphate transport, the jejunum of tenapanor-treated rats showed a decrease in NaPi-IIb expression compared with control rats, based on immunohistochemistry analysis. However, this apparently does not affect actual transcellular phosphate transport [10].

Passive diffusion of phosphate across the intestinal mucosa has been described for decades, involving passage through tight junctions between cells (Fig. 2B) [30, 31]. Tight junctions encircle the apical (luminal) end of the lateral surface of adjacent epithelial cells, corresponding to a circumferential network of tight junction strands or fibrils, forming a continuous paracellular seal between the apical/mucosal and basolateral/serosal fluid compartments [41]. Paracellular barriers have been proposed to behave as if they are lined with pores that have charge and size selectivity (Fig. 2B) [41]. These strands have subsequently been recognized as members of the claudin family of tetraspan transmembrane proteins [41, 42]. However, the extent to which tight junction function is physiologically involved in the regulation of paracellular solute transport, including that of phosphate across epithelia [43, 44], remains unclear.

As regards phosphate transport via tight junctions, the mucosa-to-serosa electrochemical gradient across the intercellular spaces plays an important role. Several reports have shown that the luminal concentration of phosphate in mice fed standard chow were in the 20 mM range, exceeding its plasma levels several fold. Similar observations were made in aspirated jejunal fluids from humans, with concentrations up to 17.5 mM [45], and concentrations of up to 11.5 mM in the distal colon of rats [32] and up to 37 mM in the distal colon of mice [30, 46]. Thus, in rodents and humans a sufficient chemical gradient exists to easily drive passive, paracellular phosphate absorption. Consequently, passive phosphate transport rates increase linearly with increasing dietary phosphorus intake and luminal phosphate concentration (Fig. 2B) [45].

As regards the electrical gradient in the intestine there is a lumen-negative transepithelial potential difference, primarily because of nutrient-coupled sodium absorption. This results in a negative luminal charge of −5 to −40 mV, thought to promote phosphate transport at least partially due to the negative charge of predominantly divalent phosphate ions (Fig. 2B) [31, 47, 48].

## MECHANISM OF NHE3 INHIBITION IN THE INTESTINE BY TENAPANOR

NHE3 activity at the apical membrane of epithelial cells is regulated through multiple pathways, including transcriptional control, protein phosphorylation, protein–protein interactions and trafficking. Tenapanor is an inhibitor of the NHE3 transporter, though its precise mechanism of action—and which aspect of NHE3 regulation it is associated with—remains unclear. Previous reports have demonstrated that NHE3 regulation is largely influenced by interactions between its C-terminal cytoplasmic tail and various cellular and structural proteins, some of which link it to the cytoskeleton network [13, 49]. In rabbit ileal brush border membranes, NHE3 operates within a large protein complex, with PDZ domain-containing scaffold proteins—including Na^+^/H^+^ regulatory factor, NHERF1 (SLC9A3R1), NHERF2 (SLC9A3R2), PDZK1(CAP70/NHERF3) and IKKPP (PDZK2/NHERF4)—playing critical roles in modulating its function [49]. Intriguingly, an interaction of the cytoskeleton network with tight junctions might modulate the paracellular permeability of several solutes [43, 44, 50]. Despite the expanding clinical use of tenapanor, the exact function of this drug and its role in the intestinal absorption of several solutes, including phosphate, remain poorly defined.

## INHIBITION OF PARACELLULAR PHOSPHATE TRANSPORT BY TENAPANOR

The mechanism by which tenapanor-induced NHE3 inhibition suppresses phosphate absorption remains unclear. Andrew J. King *et al*. of Ardelyx, the company that developed this drug, proposed that the decrease in intracellular pH (pHi) induced by tenapanor alters the structure of tight junctions, leading to reduced phosphate absorption [10]—a concept that has gained widespread acceptance. In cultured human ileal enterocyte monolayers, the authors reported a rapid change in pHi and an almost instantaneous increase in transepithelial electrical resistance (TEER) with no obvious signalling pathway or tight junction protein trafficking [10]. Based on these observations, they inferred that a direct pH-sensitive conformational change might reduce tight junction permeability via an increase in TEER, thereby decreasing paracellular phosphate transport. However, TEER is frequently used as a non-invasive index of barrier integrity, yet direct correlations with pHi are limited. TEER alone is not a reliable indicator of the dynamics of electrolyte transport; additional assays of tracer permeability or tight junction protein distribution are required to fully understand how epithelial cells respond to pH stress.

Recent studies have challenged the above hypothesis of pHi changes and TEER variations [51–55]. The authors of these reports failed to observe actual pHi changes; they rather found variations in the intensity of pH fluorescent dye used to measure pHi, suggesting that the reported differences did not necessarily indicate significant pHi shifts. They further pointed out that because pHi is tightly regulated within a narrow range to support normal cellular functions, the pHi modifying effect of tenapanor is likely modest, probably restricted to *NHE3*-expressing enterocytes [10]. The maintenance of pHi in intestinal epithelial cells is facilitated by a network of cooperating transporters and buffering systems. Among these, the Na^+^/H^+^ exchangers (NHEs) play a principal role: in murine duodenal epithelium, NHE1, NHE2 and NHE3 isoforms all contribute to pHi regulation [56, 57]. Concurrently, HCO_3_-dependent transport systems, encompassing basolateral Na^+^–HCO_3_^−^ cotransporters (e.g. NBCn1) and Cl^−^/HCO_3_^−^ exchangers facilitate intracellular buffering and expeditious recovery from acidification [58–60]. In the event of apical NHE3 inhibition the remaining mechanisms have the capacity to compensate and become activated, thus restoring pHi. The influx of HCO_3_^−^ and NHE1-mediated H^+^ extrusion may be sufficient to maintain pHi near to normal, thereby preserving epithelial homeostasis even in the presence of impaired apical proton export. Consequently, the acute effects of NHE3 inhibitors may not be equivalent to their chronic effects.

In studies utilizing jejunal villous epithelium from mouse small intestine [51] or rat duodenal loop [42], pharmacological inhibition of NHE3 with agents other than tenapanor did not alter pHi in wild-type animals either. In contrast, in the above mouse study a more acidic pHi was observed in *NHE3* knockout than in wild-type mice [51]. It is conceivable that a similar effect might be obtained if the degree of NHE3 inhibition by tenapanor were comparable to that of genetic ablation. Further investigation is needed to determine the magnitude of the pHi change in response to high doses of tenapanor.

TEER is widely accepted as a quantitative method for assessing tight junction integrity in culture models of endothelial and epithelial cell monolayers [53]. TEER values are also recognized as instantaneous measurements of ionic conductivity, providing insight into both the integrity and ion selectivity of tight junctions [54]. Although TEER and paracellular permeability are often considered to reflect similar tight junction characteristics, a study done in a renal tubular cell line has shown a functional dissociation between these two parameters [55]. Tight junctions behave as a series of diffusion barriers, which do not always remain tightly sealed but instead fluctuate between open and closed states [55, 61]. In particular, even for the same cell type, reported TEER values vary widely. Observed discrepancies can arise from multiple factors, including measurement accuracy, electrode selection and usage, temperature during measurement, medium composition, cell culture duration and passage number of the cells used in the model [53]. Therefore, caution should be exercised when asserting that an increase in TEER necessarily suppresses paracellular phosphate transport.

## TENAPANOR-INDUCED CHANGES IN LUMINAL pH AND PHOSPHATE PROTONATION STATE

Both a marked increase in luminal surface pH and in bicarbonate output into the intestinal lumen have frequently been reported not only in animals with *NHE3* deficiency but also in response to NHE3-specific inhibitors [26, 27, 62–64].

Although the terms phosphorus and phosphate are often used interchangeably, they refer to distinct chemical entities, with phosphorus being the elemental form and phosphate representing molecules containing the PO₄³⁻ ion. This distinction is important for calculating physiological balance and understanding chemical and metabolic reactivities [30, 33]. In particular, attention should be focused on the metabolism of inorganic phosphates (compounds containing Pi), which behave as weak acids in the intestinal lumen [65]. Phosphoric acid, a triprotic acid containing three ionizable hydrogen atoms per molecule, has three dissociation constants (pKa values: 2.2, 7.2 and 12.7); at physiological pH (7.4), owing to the pK₂ value of 7.2, phosphate exists in dynamic equilibrium between H₂PO₄⁻ and HPO₄²⁻, with the latter becoming dominant under alkaline conditions (e.g. pH 8.4). Accurate evaluation of the impact of electrochemical gradients on the transmembrane passage of phosphate ions requires knowledge of both their respective valence states and their effective concentrations.

Recent research has highlighted a novel aspect of the influence of lumen pH on intestinal phosphate transport—specifically, its effect on the paracellular pathway [30]. Studies have shown that compared with lower pH conditions, paracellular phosphate permeability is suppressed at higher pH values [30].

In experiments using rat jejunum, ileum, distal colon, and several intestinal cell culture models the permeabilities for Na⁺ (P_Na_), Cl⁻ (P_Cl_) and Pi (P_Pi_), as well as TEER, were measured at luminal pH values of 6.0 and 8.4 [30]. The objective was to determine whether the paracellular pathway predominantly transports one of the two phosphate ion species present under physiological conditions (H₂PO₄⁻ and HPO₄²⁻). At the respective pH values, more than 95% of phosphate exist as either monovalent ion (at a pH of 6.0) or divalent ion (at a pH of 8.4).

Permeability results are expressed as relative values (P_Na_/P_Cl_ and P_Pi_/P_Na_), as variations in transepithelial resistance influence the absolute permeability of all ions. In all tested intestinal cell culture models, monovalent Pi (H₂PO₄⁻), the predominant species at a pH of 6.0, passed through tight junctions more efficiently than its divalent form (HPO₄²⁻), predominant at a pH of 8.4. TEER remained unaffected in most cell culture models, except in T84 cells (of human colon carcinoma origin), where TEER even decreased under alkaline conditions [30].

With respect to intestinal segments, the permeability ratio P_Pi_/P_Na_ was significantly reduced at a pH of 8.4 at all sites. However, the P_Na_/P_Cl_ ratio remained unchanged in the jejunum and ileum, whereas it increased in the colon at a pH of 8.4 [30]. In that study, TEER was unaffected by alkaline pH. Based on these observations we infer that an increase in intestinal luminal pH significantly reduces phosphate transport, mainly because of its nearly sole presence as divalent ion, even when TEER decreases or remains unchanged. However, the functional characteristics and molecular mechanisms responsible for the observed selective phosphate permeability, favouring monovalent phosphate over divalent phosphate transfer, remain unknown.

**Figure 3:**
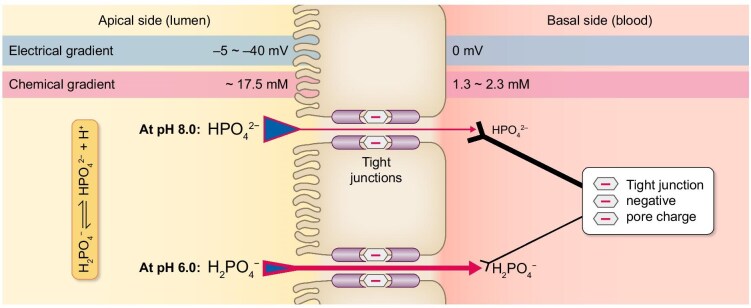
Differential paracellular permeability of monovalent and divalent phosphate ion forms across pH gradients. The luminal pH of approximately 8.0 prevailing in the colon is thought to favour the transport of divalent phosphate anions from the luminal to the serosal side. In contrast, the luminal pH of approximately 6.0 prevailing in the jejunum favours the absorption of monovalent phosphate anions (blue triangles). However, experimental evidence supports better permeability of monovalent than divalent phosphate ions under acidic conditions (red arrows). This suggests that one needs to consider an effect of the net negative electrostatic environment of the pore pathway in the formation of tight junctions (black arrowheads). The hypothetical negative pore charge at the tight junction is illustrated using red minus signs within the hexagons.

Given that the intestinal lumen maintains transepithelial potentials of approximately −5 mV in the small intestine and −40 mV in the distal colon relative to the vascular side, the divalent anion HPO₄²⁻ should theoretically exhibit greater permeability than the monovalent anion H₂PO₄⁻ due to both the electrical gradient and the difference in transepithelial phosphate concentration (Fig. 3) [47]. However, the experimental observations above indicate a preferential absorption of H₂PO₄⁻ [30]. This suggests that the negative charge of the pore pathway between tight junctions plays an important role in phosphate ion transport, inhibiting HPO₄²⁻ permeability relative to that of H₂PO₄⁻. Claudin-based channels forming the pore pathway are believed to admit only very small solutes (≤0.6 nm) and to possess a net negative electrostatic environment (Fig. 3) [66–68]. Thus, paracellular permeability in the intestine is also constrained by the size and charge selectivity of tight junction pores, which may disproportionately repel divalent anions compared with monovalent anions. As a result, H₂PO₄⁻ exhibits greater passive paracellular permeability than HPO₄²⁻. Nonetheless, the mechanisms underlying ion selectivity and permeability in paracellular pathways remain incompletely understood, and further studies are warranted to elucidate these processes.

Phosphate binds to calcium and magnesium in the intestinal lumen. Since the solubility of calcium- and magnesium-phosphate compounds decreases as luminal pH increases [69, 70], it is reasonable to assume that in response to an increase in luminal pH brought about by tenapanor the amount of soluble phosphate available for absorption is reduced. In other words, more phosphate would be sequestered as insoluble metal-phosphate complexes with Ca²⁺ and Mg²⁺, lowering free phosphate ion concentrations and ultimately impairing luminal phosphate uptake. Such an alternative mechanism of action cannot be ruled out.

## MAIN SITE OF TENAPANOR’S ACTION

The transport of phosphate in the intestinal tract has been studied extensively for decades [30, 32, 71]. However, since the first report linking tenapanor administration to phosphate transport was published [19], the focus has shifted toward identifying the primary intestinal site responsible for regulating serum phosphate levels.

The small intestine, the longest part of the gastrointestinal tract (6–7 m in humans), connects to the stomach via the pylorus and to the colon through the ileocecal valve. It consists of the duodenum, jejunum and ileum. Although electrolyte absorption, including that of phosphate, primarily takes place in the small intestine, the potential importance of phosphate secretion into the intestinal lumen should not be disregarded. In addition to pancreatic juice and bile, which enter the duodenum via the duodenal papilla, intestinal wall glands contribute substantially to chyme volume and content. Notably, all these secreted fluids contain significant amounts of phosphate [72–78] (Fig. 1). However, there are no reports that have examined phosphate concentrations in each secreted fluid of the same individual in detail. The only information available to estimate the amount of phosphate in the secretions is the difference in luminal phosphate concentrations between the small and the large intestine (Fig. 1) [30, 31].

It is noteworthy that phosphate concentration in the cecum and beyond is greater than that in the distal part of the small intestine. Luminal phosphate concentrations in the colon of rats and mice on a standard diet ranged from 14 to 40 mM, far exceeding plasma levels (2–3 mM) [30, 31, 46]. Although phosphate levels are higher in the large than the small intestine no statistical comparison of the difference has been performed [30, 31, 46]. Although several studies have demonstrated that large amounts of phosphate are absorbed in the small intestine [31, 79], the high luminal concentrations of phosphate in the large intestine can be explained by a still significant amount of phosphate remaining after absorption upstream, and by the continued absorption of water. In a study in pigs, 64% of the endogenous phosphorus output remaining at the end of the small intestine was likely reabsorbed in the large intestine [80]. Traditionally, the physiological role of the colon in phosphate absorption has been considered minor because of the increasing solidity of its contents. However, recent findings suggest that the colon serves as the final site for electrolyte and water reabsorption before fecal excretion [81]. In particular, in the distal colon, steeper electrochemical gradients are required to reabsorb NaCl and water, resulting in the formation of solid stools [67]. That phosphate is well absorbed in the colon has long been known, mainly via the paracellular pathway [82]. The use of over-the-counter saline laxatives containing huge amounts of phosphate can induce hyperphosphatemia since in presence of the high concentration gradient between gut lumen and blood phosphate is rapidly and massively absorbed. This can lead to fatal hyperphosphatemia in patients with impaired kidney function [83].

Studies in enterocyte-specific *NHE3* knockout (*NHE3IEC*-KO) mice have further emphasized colon’s role in the NHE3-induced suppression of phosphate transport [84]. In these animals, intestinal Na^+^ content was elevated across all intestinal regions, resulting in an approximately 3-fold increase in total intestinal Na^+^ content compared with that in wild-type control mice. However, intestinal phosphate content in proximal and distal small intestine did not differ between genotypes, whereas the phosphate content in the colon was approximately 1.9 times greater in the *NHE3IEC*-KO mice. Therefore, it can be inferred that the decrease in phosphate transport caused by NHE3 inhibition primarily occurs in the large intestine.

## UPREGULATED BICARBONATE TRANSPORT IN RESPONSE TO THE INHIBITION OF NHE3

As mentioned above, the valence of phosphate ions in the intestinal lumen is mainly determined by HCO_3_^⁻^ secretion, and HCO_3_^⁻^ influx into the colon plays a critical role in determining intraluminal pH [60, 85]. In the colon, a substantial portion of net Na⁺ absorption is mediated by electroneutral NaCl transport, which relies on the coupled function of two exchange mechanisms, i.e. NHE3 and Cl⁻/HCO_3_⁻ exchange. The latter is influenced by the gene product of Downregulated in Adenomas (*DRA*, SLC26A3) [86, 87]. The *DRA* gene is highly expressed in the colon and cecum, and to a lesser extent also in the small intestine [88, 89]. Another apical Cl⁻/HCO_3_^⁻^ exchanger, the putative anion transporter (*PAT1*, SLC26A6), is prominently expressed in the proximal small intestine but not in the colon. It is therefore unable to cooperate with, or substitute for, DRA in colonic anion transport [90].

**Figure 4:**
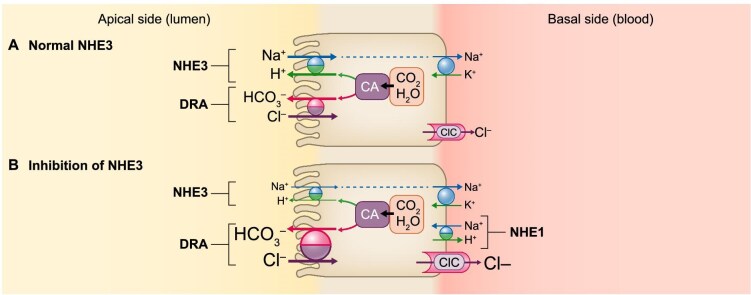
Effect of NHE3 inhibition on HCO₃^−^ transport in the colon. (**A**) Normal NHE3. Both the apical NHE3 and the Cl^−^/HCO₃^−^ exchanger (SLC26A3, also known as Downregulated in Adenomas, DRA) are prominently expressed in colonic epithelial cells. Their tandem operation plays an important role in electroneutral NaCl absorption. Carbonic anhydrase (CA) catalyses the synthesis of H⁺ and HCO₃⁻ from membrane-permeable products (CO₂ and H₂O) [86]. (**B**) Inhibition of NHE3. In the event of NHE3 inhibition, the unpaired expression of NHE3/DRA on the mucosal surface results in net colonic HCO₃ secretion and luminal alkalinization. Under these conditions, the rate of Cl^−^/HCO₃^−^ exchange exceeds the rate of Na⁺/H⁺ exchange. Excess intracellular H^+^ and Cl^−^ are suspected to be exported into the interstitial space via NHE1 [60] and poorly characterized calcium-activated chloride channels at the basolateral sites of epithelial cells [95].

Although the coordinated operation of apical Na⁺/H⁺ exchange and Cl⁻/HCO_3_⁻ exchange enables efficient NaCl absorption, an experimental study in rat intestine has shown that the proximal colon expresses abundant *NHE3* but only minimal *DRA* (0%–20% of the colon length). In contrast, the cecum and distal colon strongly express *DRA* but lack NHE3 (present in 60%–100% of the colon in rats and 75%–100% in mice). Notably, both exchangers coexist only in the middle third of the rodent colon, where their coupling could maximize NaCl absorption [86]. The study demonstrated that *NHE3* and *DRA* exhibit distinct expression patterns throughout the colon. In the distal colon, similar to the cecum, high levels of *DRA* but negligible amounts of *NHE3* are expressed, consistent with previous reports that this segment can rapidly secrete HCO_3_^−^ in a manner that is electroneutral and coupled to Cl^−^ absorption (Fig. 4) [91–94].

In addition, a genome-wide analysis of colonic gene expression in *NHE3*-deficient mice revealed upregulation of several ion transporters, including DRA and PAT1 (SLC26A6), which are involved in Cl^−^/HCO_3_^−^ exchange [95]. Thus, the hypothesis was made that under conditions of NHE3 inhibition the upregulation of DRA increases bicarbonate secretion and Cl^−^ absorption, and these changes may represent adaptive responses to maintain bicarbonate secretion and acid–base balance in the absence of normally functioning NHE3 (Fig. 4) [95]. Because NHE3 inhibition results in the loss of large amounts of NaCl from the intestine, complementary mechanisms are required to absorb Cl^−^, especially as the amount of Cl^−^ remaining in the lumen limits the amounts of Na⁺, K⁺ and water that can be absorbed. Consistent with previously published observations, the expression of *DRA* was increased 2.17-fold in *NHE3*-deficient mice (Fig. 4) [87, 95].

Studies in global *NHE3* knockout mice further support this notion, showing that intestinal luminal pH was significantly elevated in the small intestine, cecum and colon compared with that in control mice, with the greatest increase observed in the cecum, followed by the colon and small intestine [26]. Additionally, tenapanor has been shown to increase alkaline output in the colon and jejunum in association with the inhibition of fluid absorption in mice [63]. Therefore, it can be inferred that NHE3 inhibition contributes to increased bicarbonate secretion in the colon.

## CONCLUSION

Tenapanor, originally developed as an NHE3 inhibitor for the treatment of IBS-C, was subsequently found to achieve a clinically meaningful reduction of increased serum phosphate levels among patients on dialysis by inhibiting intestinal phosphate absorption. However, the underlying mechanisms of action remain incompletely understood. In this review we propose a tentative explanation involving an increase of luminal pH in the colon secondary to enhanced bicarbonate secretion caused by the inhibition of NHE3. At alkaline pH, the major shift in luminal phosphate ion speciation from monovalent to divalent form leads to a reduction of intestinal phosphate absorption due to the electrochemical properties of the paracellular pathway.

Although the paracellular permeability of divalent anions is theoretically superior to that of monovalent ions in the presence of lumen-negative transepithelial potential, recent evidence suggests that divalent phosphate species are less efficiently absorbed, with anion paracellular permeability being reduced at high pH values. We therefore hypothesize that a net negative electrostatic environment within the pore pathway of tight junctions selectively impedes divalent phosphate transport. This physicochemical mechanism is supported by the observation that tenapanor does not alter tight junction protein expression, suggesting a nonstructural basis for reduced permeability.

Further investigations are warranted to determine to which extent the electrostatic properties of the paracellular pathway contribute to the phosphate-lowering effect of tenapanor. Clarifying this mechanism may enhance our understanding of intestinal phosphate handling and inform future therapeutic strategies for hyperphosphatemia in patients with CKD.

## Data Availability

No new data were generated or analysed in support of this research.
